# Modulation of the P-Glycoproein-Mediated Intestinal Secretion of Glibenclamide: *In Vitro* and *In Vivo* Assessments

**DOI:** 10.4103/0975-1483.71632

**Published:** 2010

**Authors:** P Srirangam, Sagar J Vidya

**Affiliations:** *Department of Pharmacology, Vaagdevi College of Pharmacy, Warangal, AP, India*

**Keywords:** P-glycoprotein, glibenclamide, carbamazepin

## Abstract

The everted gut sac method was used to assess the role of the P-glycoprotein (P-gp) on the intestinal secretion of glibenclamide, a prototype of drug used to treat diabetic mellitus. The study included the evaluation of a P-gp modulator carbamazepine used at equimolar doses in the rat. Furthermore, the influence of carbamazepine on the disposition kinetics of glibenclamide in plasma was characterized. For the *in vitro* experiments, ileal sacs were incubated with glibenclamide in the presence or absence of carbamazepine. In the *in vivo* experiments, albino rats of either sex were randomly allocated to two groups (*n* = 6) and oral treatment with glibenclamide (3.6 mg/kg), alone and coadministration with carbamazepine (90 mg/kg). Blood samples were collected at an interval of 1, 2, 4, 6, and 8 h, respectively. Glibenclamide concentrations in both *in vitro* and *in vivo* samples were estimated by a sensitive RP-HPLC method. The rate of glibenclamide accumulation in the intestine wall of everted sacs was significantly lower after its incubation with carbamazepine when compared to glibenclamide alone treated. In the agreement with the *in vivo* and *in vitro* experiments, the presence of carbamazepine induced an enhancement in the concentrations of glibenclamide in plasma and gastrointestinal tract. The results obtained in this study, both under *in vivo* and *in vitro* conditions confirm the relevance of P-gp-mediated transport to the intestinal secretion of glibenclamide.

## INTRODUCTION

P-glycoprotein (P-gp) is a transmembrane protein, associated with a phenotype of multidrug resistance to certain anticancer drugs in mammalian cancer cells, that is able to pump a broad range of structurally and functionally unrelated compounds out of the cell by an ATP-dependent process. New *in vitro* models have been developed to predict *in vivo* P-gp activity, in which Caco-2 cell monolayers are widely used to estimate transepithelial passage of different P-gp substrates.[1,2] However, the correlation between this *in vitro* model and *in vivo* studies is rather poor. The use of everted gut sacs has been proposed as a new *in vitro* model for quantification of P-gp-mediated intestinal efflux for various drugs.

This study is planned to evaluate the safety of glibenclamide (an anti-diabetic drug) therapy in the presence of carbamazepine (a prototype drug used to treat painful diabetic neuropathy) in healthy rats. The interaction mechanisms in preclinical studies help to avoid adverse effects when this particular combination was administered to patients suffering with painful diabetic neuropathy.

## MATERIALS AND METHODS

### Drugs and chemicals

Glibenclamide and carbamazepine are the gift samples from Alka Pharmaceuticals (Hyderabad, India). D-PBS (Sigma-Aldrich, USA), the HPLC grade methanol and acetonitrile of Qualigens Fine Chemicals, Mumbai were procured from local chemical suppliers. All other chemicals used were of analytical grade.

### Animals

Experiments were performed with albino rats procured from Mahaveera Enterprises (Hyderabad, AP, India), weighing between 180 and 210 g. The animals were housed in colony cages (four per cage) under conditions of standard lighting, temperature (22 ± 1°c) and humidity for at least 1 week before the beginning of experiment, to adjust to the new environment and to overcome stress possibly incurred during transit. During this period, they had free access to food and water. The experiments were planned after the approval of Institutional Animal Ethical Committee (IEAC), Vaagdevi College of Pharmacy, Warangal, AP, India.

## METHODS

### *In vitro* experiment

The experimental procedure was performed by modified method of Yumoto *et al*.[3] The whole small intestine was isolated and flushed with 50 mL of ice-cold saline after killing the animal by overdose of pentobarbital. The small intestine was divided into duodenum, jejunum, and ileum. Ileul segment was everted, and a 10-cm long sac was prepared (i.e., P-gp is more expressive in ileum). About 1 mL of glibenclamide solution (1 mg/mL) was introduced into the everted sac (serosal side) and both ends of the sac were ligated tightly. The sac-containing glibenclamide solution was immersed into 30 mL of Dulbecco’s phosphate buffer solution (D-PBS) containing 25 mM of glucose, and the same concentration of carbamazepine in DMSO was introduced into the mucosal side and the solution was prewarmed and oxygenated throughout the experiment. The transport of glibenclamide from serosal to mucosal surface across the intestine was measured by collecting samples from the mucosal medium periodically at different intervals 0, 10, 20, 30, 60, 90, and 120 min and all samples were conserved at –20°C until analysis. Using this medium, the transport of glibenclamide in the absence (control) and presence (test) was measured.

### *In vivo* experiment

Albino rats of either sex were randomly distributed into two groups of six animals in each group. Before experiment, all animals were fasted for 18 h and water *ad libitum*, water was withdrawn during experiment. After collection of initial blood samples, drugs were administered in the following order. Animals in Group A (GLB alone) received GLB at 3.6 mg/kg by oral route and rats in Group-B (GLB + CAR) received CAR (90 mg/kg) before 1 h of GLB (3.6 mg/kg) treatment by oral route. The blood samples were collected from orbital sinuses using heparinized capillaries into a microcentrifugation tubes contain anticoagulant at 1, 2, 4, 6, and 8 h after treatment. Plasma was separated by centrifugation and stored at −20°C until further analysis.

### Bioanalytical method

Glibenclamide concentrations from *in vitro* and *in vivo* samples were determined with a validated high-performance liquid chromatography (HPLC) method. Briefly, the HPLC system consisted of a Waters 717 plus Autosampler (Waters Co., Milford, MA, USA), a Waters 501 pump (Waters Co), and a 785 UV Detector (Applied Biosystems, Foster City, CA, USA) operated at 253 nm. The stationary phase was a Waters Symmetry C18 column (250 mm × 4.6 mm, 5 μm, Waters Co.). The mobile phase used was 25 mM sodium phosphate/acetonitrile (65:35, v/v, pH = 3.5) at a flow rate of 1.0 mL/min. Glibenclamide and an internal standard (glipizide) were isolated from plasma by liquid-liquid extraction with methanol. The organic phase was separated and evaporated, and the remaining residue was reconstituted with 250 μL of mobile phase before applied to the HPLC system. The method was validated and found to be linear over the concentration range of 0.1–10 μg/mL. Using a linear-weighted least squares regression, the lower limit of quantitation (LLQ) was 0.1 μg/mL. The intra-assay and inter-assay coefficients of variation (%CV) for the three quality control standards (0.25, 2.00, and 8.00 μg/mL) were ≤10.2% and 4.9%, respectively.

### Pharmacokinetic analysis

The pharmacokinetic parameters of glibenclamide were calculated using a sophisticated tool known as Win Nonlin (4.1) and the parameters includes half-life (*t*^1/2^), clearance (Cl), volume of distribution (*V*_d_), *C*_max_, *T*_max_, and area under the concentration-time curve (AUC).

### Statistical analysis

Data were expressed as mean ± standard deviation (SD). The significance was determined by applying Student’s paired *t*-test.

## RESULTS

### *In vitro* experiments

**Figure 1:**
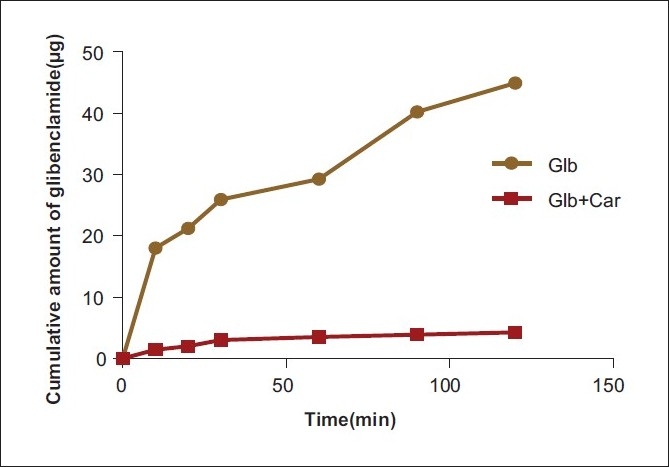
Transport of glibenclamide from serosal to mucosal side in the everted ileum of the healthy rat in presence and absence of carbamazepine (*n* = 6)

**Figure 2:**
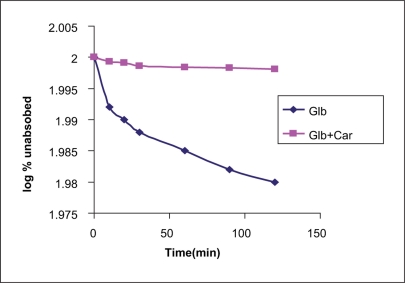
Percentage of glibenclamide remaining in the everted ileum of healthy rat at different time points in the presence and absence of carbamazepine (*n* = 6)

The time course of glibenclamide transport across the everted ileum of healthy rats was shown in Figure 1. The percentage of glibenclamide remain present in ileum are shown in Figure 2, and results of carbamazepine inhibition at different time points are given in Table 1.

**Table 1:** Glibenclamide (μg/mL) transport in the everted ileum of healthy rat in the presence and absence of carbamazepine

Time (min)	Glibenclamide-treated rats Mean ± SD	Glibenclamide and carbamazepine-treated rats Mean ± SD	*P* value
10	18.04 ± 1.6	1.44 ± 0.35	***
20	21.22 ± 1.799	2.01 ± 0.269	***
30	25.93 ± 0.4326	3.02 ± 0.627	***
60	29.29 ± 2.133	3.52 ± 0.47	***
90	40.21 ± 3.003	3.88 ± 0.345	***
120	44.92 ± 1.27	4.28 ± 0.198	***

^*^Significant at *P* < 0.05

^**^significant at *P* < 0.01

*** significant at *P* < 0.001 compared to glibenclamide control

### *In vivo* experiments

**Figure 3:**
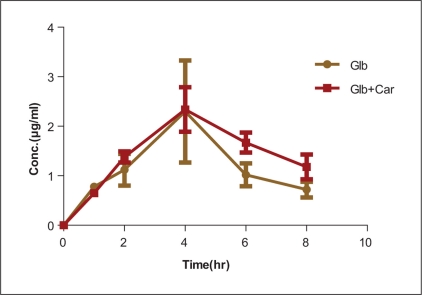
Mean ± SD plasma concentration–time profile of glibenclamide (3.6 mg/mL) following pretreatment with carbamazepine (90 mg/kg) by oral administration in healthy rats (first day)

The plasma glibenclamide levels and pharmacokinetic parameters of glibenclamide such as AUC, T1/2, clearance, *V*_d_, *C*_max_, and *T*_max_ were altered significantly when comparing the results between GLB alone and combination group (GLB + CAR), and the results are shown in Tables 2, 3 and Figure 3, respectively.

**Table 2:** Mean plasma glibenclamide concentration (μg/m) before and after oral administration of carbamazepine in healthy rats (*n* = 6)

Time (h)	GLB	GLB + CAR (First day)
0	0.00 ± 0.00	0.00 ± 0.00
1	0.78 ± 0.05	0.65 ± 0.04***
2	1.12 ± 0.32	1.38 ± 0.11
4	2.30 ± 1.03	2.34 ± 0.45
6	1.02 ± 0.23	1.67 ± 0.20***
8	0.72 ± 0.16	1.18 ± 0.25***

*** Significant at *P* < 0.001 compared to glibenclamide control

**Table 3:** The comparison of pharmacokinetic parameters of glibenclamide (3.6 mg/mL) following pretreatment with carbamazepine (90 mg/kg) by oral administration in healthy rats (*n* = 6)

Parameter	GLB	GLB + CAR (First day)
AUC (μg/mL/h)	12.96 ± 4.88	20.95 ± 3.61***
K_HL (h^−1^)	2.24 ± 0.04	2.848 ± 0.24**
CL_F (mL/h)	42.31 ± 12.37	24.51 ± 4.51*
*T*_max_ (h)	3.24 ± 0.06	4.10 ± 0.34***
*C*_max_ (μg/mL)	1.46 ± 0.51	1.86 ± 0.24
V_F (mL)	235.51 ± 45.82	147.73 ± 21.98**
				

* Significant at *P* < 0.05

** significant at *P* < 0.01

*** significant at *P* < 0.001 compared to glibenclamide control

## DISCUSSION

The pharmacological effects of a given drug compound are highly influenced by its pharmacokinetic behavior. Physicochemical properties are critical to the absorption, distribution, metabolism, and excretion of different xenobiotic compounds. However, it has now become apparent that different transport proteins play an important role in regulating the kinetic disposition of several drugs.[4]

In addition, clinically relevant drug interactions may occur after the concomitant administration of various compounds. The goal of the interaction studies between P-gp substrate and modulator has been addressed to identify the pharmacokinetic consequences and to predict the clinical outcome.

Several approaches are available to assess *in vitro* role of the specific proteins involved in the intestinal drug transport processes. However, the inference from the quantitative information obtained in the *in vitro* models to the *in vivo* situation may be limited. Thus, the prediction of the absorption process for a given P-gp substrate from *in vitro* assays could not be correlated to the *in vivo* results obtained when coadministration with a P-gp modulator is performed.[5] This outcome could be due to differences in the length of drug exposure, drug concentrations, concomitant induction of metabolic pathways, and extent of P-gp inhibition.[6]

Previous *in vitro* studies reported that GLB was actively secreted by multidrug-resistant tumor cells and by cells transfected with the gene coding for P-gp in the mouse. In addition, Barthe *et al*.[7] described the everted gut sac as a simple method to study intestinal absorption of digoxin in the presence or absence of verapamil or quinidine, obtaining a high correlation with the *in vitro* Caco-2 cells[8] and *in vivo* methods using knockout mice. The results of the current trial confirm that the everted sac technique is a useful *in vitro* model system for studying the P-gp-mediated efflux of GLB. The satisfactory results obtained with the use of the everted sac model to assess the P-gp-mediated modulation of GLB transport may be relevant to achieve reproducible results between the *in vitro* and *in vivo* assays.

The GLB concentration profiles measured in the ileal wall was markedly modified by the presence of CAR. The GLB tissue accumulation at 60 min of incubation in the presence of CAR was 20.1-fold higher compared with the incubation of GLB alone. Therefore, the rate of GLB accumulation in the intestinal wall was significantly higher in the presence of CAR. Thus, these results demonstrated that the everted sac technique was useful to compare the P-gp inhibition by modulators like CAR from different generations used at equimolar doses.

Interestingly, the results reported in our *in vitro* experiments were directly related to the observed *in vivo* changes in the pharmacokinetic behavior of GLB after coadministration with CAR [Table 3]. This is a relevant issue, considering that in previous studies, it was not always possible to reproduce *in vivo* results observed in *in vitro*. For instance, where as ketoconazole seemed to be the most valuable tool for increasing the intracellular quantity of [^14^C] moxidectin in rat hepatocyte cell cultures, the compound had no effect on the *in vivo* pharmacokinetics of moxidectin in lambs.[9–14]

## CONCLUSION

The results from the studies reported here indicate that the plasma disposition of GLB was strongly affected by P-gp activity. In addition, we have demonstrated that GLB disposition kinetics in the intestinal tissues was significantly modified by the presence of CAR, under both *in vivo* and *in vitro* conditions, which confirms the relevance of this cellular transporter on the intestinal secretion of GLB. The characterization of the intestinal elimination pathway for GLB would be of therapeutic significance and the high GLB concentrations could be available at the target tissues, and which improving its clinical efficacy.
